# A study on the mode choice of large-scale households’ farmland transfer-in in rural China: Based on the economic analysis paradigm of transaction costs

**DOI:** 10.1371/journal.pone.0287022

**Published:** 2023-10-17

**Authors:** Guoping He, Taofen Xiao

**Affiliations:** 1 School of Economics, Hainan University, Haikou, Hainan, China; 2 Management School, Hainan University, Haikou, Hainan, China; Szechenyi Istvan University: Szechenyi Istvan Egyetem, HUNGARY

## Abstract

Regarding the mode choice of farmland transfer, the existing literature have more examined the choices between market-based transfer (spontaneous transfer) and government or village committee-led transfer, and between formal contract and informal contract. However, the question that how the two parties choose among various specific transfer modes has not attracted extensive attention of scholars. Based on contract theory and transaction cost economics, this paper uses the public samples of the third national agricultural census data to investigate how large-scale households choose among the specific transfer modes when transferring into farmland, like the transfer of the contracted management right (TCMR), lease and shareholding. The findings of this paper are as follows. Firstly, with the increase in the transfer-in area and the education level of the household head, the probability of choosing lease and shareholding increases relatively, but the latter rises faster. Secondly, compared with large-scale farmers whose aim is to plant crop, the probability of those who transfer into farmland for gardening and forestry operation choosing shareholding has increased significantly. Thirdly, the age of the household head and the number of household laborers have an interactive effect on the mode choice of transferring into farmland. In addition, the mode choice of large-scale households’ transferring into farmland is also significantly affected by environmental factors such as local topography, irrigation, traffic conditions, industrial structure, and social security development. Therefore, adhering to the parties to choose the mode of farmland transfer independently is crucial. The government and rural grassroots organizations should help the parties to understand the characteristics, adaptability and supply and demand of different modes, and help the parties to explore the most economical mode. The contribution of this paper is that it expands the study of the transfer of rural land rights to the choices of specific transfer modes, and partially reveals the rule of choices, which provides a reference for the parties to choose the most efficient transfer mode under different conditions and for the government and rural grassroots organizations to play a role.

## 1. Introduction

Since the implementation of the household contractual system in rural China in the 1980s, farmland transfer and moderate scale management have occurred and become popular, and diversified transfer modes like the exchange of contracted land, the transfer of contractual management rights (TCMR), substituting farming, subcontracting, lease, “Reverse Renting and Subcontracting”, shareholding and land trust have also emerged. This is due to the change of family population and labor force and the difference of farming ability; especially with the development of industrialization, urbanization and modern agriculture, a large number of rural labor force enter the industry and commerce, and thus new agricultural management entities such as, family farms, farmers’ cooperatives and agricultural enterprises have emerged in large numbers. By 2020, the areas of transfer of contracted farmland had reached nearly 37.64 million hectares, accounting for 36.15% of the contracted (This Information is from the Ministry of Agriculture and Rural Affairs of the People’s Republic of China: http://zdscxx.moa.gov.cn:8080/nyb/pc/search.jsp). Farmland transfer plays an important role in the revitalization of rural industry, agricultural modernization, and rational utilization of land, scale management, as well as the improvement of land output rate, labor productivity and resource utilization rate [1–4]. The adaptability of the modes of farmland transfer to the characteristics of the subject and object and the environment is an important factor influencing the performance of transfer. Therefore, to study the mode choice of farmland transfer makes great senses. However, the problem about how to choose among various specific transfer modes in the transfer market has been rarely studied (see Part 2). Taking the land transfer-in of large-scale households as an example, this paper analyzes how they choose among three basic transfer modes like TCMR, lease and shareholding.

The innovations of this study are twofold. First, it expands the research field of rural land transfer, turning the focus of the research about farmland transfer to the mode choice of specific transfer. Second, this study regards various modes of land right transfer as different forms of contract, and applies the analysis paradigm of transaction cost economics to make an analysis on the mode choice for large-scale households’ farmland transfer-in. Meanwhile, the findings partially reveal the law of the parties that transfer the rural land rights to choose the transfer mode, which provides a reference for the parties to choose the most efficient transfer mode under different conditions, and also for the government and rural grassroots organizations to play a role.

The remaining part of the paper is structured as follows. Section 2 gives the details of the existing literature. Next, section 3 covers the theoretical framework, whereas section 4 shows the data sources, variable setting and statistical description of samples. Section 5 provides the empirical methodology and the analysis outcomes. Section 6 is the conclusion and section 7 is policy recommendations. Finally, section 8 presents the contributions, limitation and expectation.

## 2. Literature review

### 2.1 Review on farmers’ decisions about land transfer-out or transfer-in and transfer scale

Several studies have been undertaken in the past to investigate the decision-makings of whether farmers transfer land in or out of and the transfer scale. These decisions first depend on their comparative advantages in agricultural management or non-farm employment, transaction costs and risks of land transfer. However, the comparative advantage of farmers depends on their labor force, land resources, environmental conditions of agricultural operation and non-agricultural employment opportunities and other factors. Most studies have demonstrated that farmers’ labor force and health status have a negative impact on land transfer-out [5, 6], and a positive effect on land transfer-in [7, 8]; farmer emigration and rural labor transfer are conducive to land transfer [9, 10]. Specifically, the age of farmers has a negative [8] but decreasing influence on land transfer-out [9]. For elderly farmers, age has a positive impact on land transfer-out due to physical decline [6]. In addition, Some studies recommend that the more educated farmers are more likely to choose non-farm employment, which has a positive effect on land transfer-out [11]. However, some other researchers find that the higher the education, the stronger the operation capability, and thus the more likely to transfer into land for large-scale operation, which has a negative influence on land transfer-out [9].

Generally speaking, non-farm employment, part-time farming, non-farm income and its proportion, and the transfer income from government reduce farmers’ dependence on land and thus have a positive impact on land transfer-out [6, 9, 11, 12]. There are also studies concluding that the probability of farmers’ land transfer-out increases in the order of farmers mainly engaged in agriculture, farmers living only by farming, farmers mainly engaged in non-farm and non-farmers; the proportion of the area of transfer-out in the area of the household’ land shows a U-shaped relationship with the degree of concurrent employment [5].

Some scholars have discussed the impacts of land resources and other social resources on land transfer. The unit transaction cost of land transfer-out decreases with the increase of land owned by farmers, which has a positive impact on transfer-out. But the scale effect gradually appears and the possibility of transferring into land for large-scale operation gradually increases when the land owned by farmers increases to a certain scale, which has a negative impact on land transfer-out [6]. Therefore, farmers’ land resources are inversely U-shaped with the land transfer-out [11]. Besides, the willingness to further transfer into land becomes lower when farmers’ land meets or exceeds the requirements of scale economy. Thus, farmers’ land resources also show an inverted U-shaped relationship with land transfer-in [7]. The more fragmented the land, the less convenient the operation is, the worse the quality of agricultural land and thus the more likely to transfer out of land [11, 13]. Furthermore, farmers’ financing opportunities have a positive impact on land transfer-in [7] and a negative impact on land transfer-out [9]. Village cadres have more social resources and are more likely to transfer into land for large-scale operation than ordinary farmers, and thus have a negative impact on land transfer-out [6] and a positive effect on land transfer-in [7]. The information, trust and reputation network of farmers reduce transaction costs of land transfer, which has a positive impact on farmers’ participation in the land market [14].

In addition, some studies have examined the impacts of environmental conditions such as the distance from cities and towns, terrain and infrastructure on land transfer. Generally speaking, the farther away from the town, the worse the topography conditions, and thus the higher the farmers’ willingness to transfer out of land [8]; but for the rural elderly, the farther away from the town, the worse the terrain, the less opportunities to earn money, and thus the more dependent on land for pension, which has a negative impact on land transfer-out [6]. The better the transportation and other infrastructures, the stronger the attraction of land to capital, the greater the demand for land, and the higher the transfer price, which has a positive impact on farmers’ land transfer-out [5, 9, 11], and a negative impact on land transfer-in [7]. In addition, the higher the level of local economic development, the more opportunities for non-agricultural employment. Therefore, it has a positive effect on farmers’ land transfer-out and a negative impact on land transfer-in [7].

The transaction costs of land transfer could be reduced by the improvement of land rights, the formality and convenience of transactions, the development of land market intermediaries, the participation of rural collective organizations, and the transaction experience and land reserves and so forth, which is conducive to developing land market [14–21]. But the adjustment of village collective to the contracted land is adverse to land transfer [22]. Besides, the development of rural social security diminishes farmers’ dependence on land and has a positive impact on land transfer-out [5, 8]. Agricultural technology training and subsidies for large-scale operation have an important impact on farmers’ land transfer [13, 23]; basic farmland protection policy is unfavorable to land transfer [24]. Moreover, the motivation of farmers’ land transfer is not entirely profit maximization; the irrational factors such as the attachment to land, agricultural fun and conservative tendency have an important impact on farmers’ behavior to transfer land. Innovative and optimistic farmers are more likely to participate in the land market [25].

### 2.2 Review on the mode choice of farmland transfer

With regard to the mode choice of rural land transfer, the previous literature have more investigated the choice between market-based transfer (spontaneous transfer) and government or village committee-led transfer, and between formal contract and informal contract. Some studies explore that there are more spontaneous transfer among farmers in land transfer in non-suburban rural areas; however, in suburban rural areas, the government or village committee-led circulations occur more frequently [26]. Driven by political achievements [27], government-led or village committee-led land transfer easily breeds forced transfer and rent-seeking [28], distorts land resource allocation and reduces allocation efficiency [29]. Therefore, some scholars advocate promoting market-oriented circulation [28, 29]. Nevertheless, other scholars have emphasized that government or village committee dominance decreases transaction costs and risks of land transfer and is conducive to maintaining the agricultural purposes of land [30].

Farmland property rights and its supporting system, the relationship between the parties to the transaction and the participation of government or village collective, land use and so forth have an important effect on the choice of the contract form of land transfer (formal contracts and informal contracts). The signing of formal contracts are significantly promoted by the confirmation of contracted land rights, the establishment of land transfer dispute mediation institutions [31], the constraints of the transfer system, and the participation of village collectives [32]. However, the joint relationship between the parties and the uses of land for food crop cultivation significantly promote oral contracts [32]. Nguyen, Rigg [10] demonstrates that the lease or exchange of agricultural land mainly relies on oral agreements which depend on social capital and trust networks since the relevant system has been unestablished in Vietnam during the reform period.

Besides, some studies examine the choice of price of land transfer and contract flexibility. For instance, the land rights [33] and its risks [34], transaction costs [35], economic development level, land market maturity [36], human feelings [37], and emotions about land [38] have an important impact on the price of land transfer. Land fragmentation, poor land quality, poor irrigation conditions and marginalization of mountainous areas are key factors causing unrewarded land transfer [39]. Because farmers’ decisions on land transfer are based on the principle of satisfaction, there is a deviation between the actual price of land transfer and the market price. Generally speaking, this deviation is relatively small in mature market and economically developed regions [36]. Tretiak & Moskalenko etc. [40] studies land transfer under the land share system in Ukraine and demonstrates that transfer price is artificially formed at the standard monetary valuation level of land rather than by supply and demand. Additionally, Yang [41] discusses the flexibility of land lease contracts and the choice of rent payment, and indicates that lease agents increase the flexibility of contracts and reduce rents; social proximity helps non-stranger entrepreneurs obtain low-flexibility contracts and increase rents.

Some researchers have explored how farmers choose among specific land transfer modes. They show that those choices are mainly affected by farmers’ age, education level, risk preference, land endowment, as well as non-agricultural employment, family income and its stability. For instance, Wang and Gu [42] examines households’ selection in several modes of cultivated land allocation, like self-farming, substituting farming, renting and shareholding. They find that farmers with higher education and family income are more likely to choose lease and shareholding, and risk lover are more likely to choose shareholding. Wang & Yang [43] confirms that part-time farming has an important influence on the mode choices of farmland transfer in Chongqing. The household heads’ age has a positive effect on farmers living only by farming and farmers mainly engaged in agriculture choosing substituting farming, and farmers living only by farming and farmers mainly engaged in non-agriculture choosing subcontracting and leasing, while has a negative influence on farmers mainly engaged in non-agriculture and non-farmers choosing TCMR. Farmers’ land endowment only significantly affects mode choices of farmland transfer of farmers living only by farming and farmers mainly engaged in agriculture. The distance of non-farm employment only significantly influences mode choices of farmland transfer of farmers mainly engaged in non-agriculture and non-farmer, while the stability of non-farm employment only has a positive impact on non-farmers’ choice of TCMR. Land ownership also affects mode choices of transfer [44]. Alves & Maeda etc. [45] discovers that land ownership makes it easier to sell land, but has no effect on the convenience of leasing and bequests in Brazil. Ali & Deininger [46] studies the large farms in Zambia and shows that land ownership reduces participation in the rental market. Furthermore, some researchers examine that subcontracting, lease and shareholding show certain spatial rules [47]. Lease has always been the most common for farm integration among the various specific modes of farmland transfer [48]. Therefore, the existing literature are more about research on land leasing. Karwat-Wozniak & Buks [49] discusses that land leasing in Poland occurs mainly between neighbours; neighborhood leasing will be the basic way to provide land to commercial farms with the depletion of unallocated treasury land resources and farmers’ dependence on heritage.

Moreover, some scholars analyze the choice and change of fixed rent contract and shared tenancy contract. Barzel [50] argues that the costs of supervising the use and improvement of land under fixed rent contracts are high. The costs of supervising labor and output are high under the shared tenancy contract, thus, its transaction costs seem to be higher than that of fixed rent contract. But shared tenancy contract can disperse risks. people will choose shared tenancy contract when its higher transaction costs can be compensated by the benefits of risk diversification [51]. Sun & Yang [52] explains the changes of land tenancy contract in the Republic of China from the perspective of transaction costs, and believes that the replacement of shared tenancy contract and the permanent tenancy system by the short-term fixed rent contract is due to the rise of landlords’ moving to towns and agencies.

As mentioned above, regarding the mode choice of farmland circulation, the previous literature have mainly examined the selection between market-based transfer (spontaneous transfer) and government or village committee-led transfer, as well as between formal contract and informal contract. However, the problem that how the transferor and transferee in farmland market choose specific circulation modes such as TCMR, lease, and shareholding, namely the circumstance that the circulation subjects with different characteristics may choose which specific modes under what conditions has not yet attracted extensive attention of the existing literature.

Based on contract theory and transaction cost economics, this paper adopts the public samples of the Third National Agricultural Census, and takes the case that large-scale households transfer into cultivated land to analyze how they select among three basic circulation modes of TCMR, lease and shareholding, focusing on the impacts of circulation areas, uses and family characteristics on the mode choices of circulation.

## 3. Theoretical framework

As mentioned above, in China, there are various circulation modes in cultivated land transfer, such as exchanging contracted land, the transfer of contractual management rights (TCMR), substituting farming, subcontracting, lease, “Reverse Renting and Subcontracting”, shareholding, land trust, auction and sale of land management rights during realizing mortgage of that rights. But from the perspective of land transfer-in and expanding management scale, only TCMR, lease and shareholding (namely absorbing others to share with the land management rights) are three basic circulation modes (The reasons are as follows. Subcontracting, as a special case of lease, refers in particular to the lease between members of the same collective [59]. “Reverse Renting and Subcontracting” means that the township government or village collectives lease the contracted land of households, and then subcontract those land to the land operator. For land operators, their mode of transferring into land is still lease or subcontracting, but they make transactions with township government or village collective directly. Land trust refers to that households entrust land management rights to trust institutions to transfer, mainly including rent (namely leasing trust), or selling management rights by bidding and auction (namely selling trust) [67]. But the survey found that agricultural enterprises and farmer cooperatives were the main participants in bidding and auction in practice and large-scale households mainly chose lease to transfer into land from trust institutions. Whereas, in exchanging contracted land, both sides do not expand the land management scale because they only exchange their contractual management rights between members of the same collective. Thus, exchanging contracted land is not the basic way for large-scale households to transfer into land and expand management scale. Substituting farming is farming other’s contracted cultivated land entrusted by others and mostly is informal contracts, with narrow transaction scope mainly appearing between relatives or neighbors, which plays a very limited role in the allocation of rural land resources and scale management. The transfer of land management rights also includes realizing the mortgage in the financing guarantee of land management rights when the debtor fails to perform the due debt or when the parties agree it is necessary, mainly through valuation, auction and sale of management rights. Obviously, this situation, attached to the financing guarantee of land management right, only appears under the above specific circumstances, and thus it is also not the main way for land operators to transfer into cultivated land and expand the management scale.). Next, this paper mainly discusses how large-scale households select among three basic modes of TCMR, lease (Lease discussed in this paper includes large-scale households directly leasing other households’ contracted land, subcontracting (a special case of lease), and indirectly leasing or subcontracting households’ contracted land in “Reverse Rent and Subcontract”, as well as indirectly leasing the households’ contracted land through trust institution.) and shareholding when they transfer into cultivated land.

According to contract theory, any contractual arrangement is the result of people’s selection in order to reduce transaction costs. Under “the Separation of Three Rights”, large-scale households’ mode choice of transferring into cultivated land can be explored on the issue of saving transaction costs. First, Coase [53] analyzes the costs of market transaction and indicates that it includes all costs of finding relative prices, negotiation and contracting in transaction, and other costs of using price mechanism. In 1960, he proposed the concept of market transaction costs, generalized transaction costs and analyzed the organizational costs of enterprises and governments, as well as the costs of defining and executing rights [54]. Subsequently, after the 1970s, Scholars tended to consider contracts as the structure of transactions, regard organizational system issues as contractual issues, and understand transaction costs in terms of contracts. For instance, in terms of contractual process, Dahlman [55] summarizes transaction costs into information costs, costs of bargaining and decision-making, costs of supervision and implementation. Similarly, Dietrich [56] defines transaction costs as the costs of investigation and information, negotiation and decision-making, and policy formulation and implementation. Additionally, Williamson [57] classifies two kinds of factors that affect or determine transaction costs. One kind is transaction characteristics, which mainly concern the characteristics of market environment and the technical structure of transactions, including asset specificity, uncertainty, number of potential counterparties and transaction frequency. The other one is human factor, namely human bounded rationality and opportunism tendency. The followings are specific descriptions regarding these factors. First, among them, the asset specificity (namely the specificity of asset itself, asset location specificity, human capital specificity, etc.) is the most important. The stronger asset specificity, the more specific quasi-rents, which increases serious opportunistic behavior, and thus the more likely both transaction parties are to establish a stable and persistent contractual relationship. Second, the number of latent counterparties is an important factor influencing transaction costs. When there are many counterparties, the contract is easy to be achieved and implemented as both sides of the transaction are less dependent on each other and the search cost is low. In addition, competition plays a crucial role in inhibiting opportunistic behavior, so the traders pay less to prevent opportunistic behavior. Whereas, as the number of trading counterparties is small, search costs increase and the probability of reaching successful contract negotiation decreases, and thus costs of reaching transaction increase. In addition, the non-monopolist party has a great dependence on the monopoly, and the possibility of opportunistic behavior of the monopoly increases greatly, thus transaction costs increases. Third, the influence of transaction frequency on transaction costs is mainly manifested in that in contrast to the one-time transaction, the frequent transaction is easier to compensate the costs of establishing and operating transaction regulation structure and relatively reduces the transaction costs. Additionally, the uncertainty includes environmental uncertainty and uncertainty caused by opportunistic behaviors such as strategic concealment and distortion of information caused by both parties. Furthermore, bounded rationality increases the cost of searching, waiting and bargaining for transactions. Due to the bounded rationality of individuals, the complexity and uncertainty of external environment, as well as the incompleteness and asymmetry of information, the contract parties or arbitrators cannot confirm or observe everything and thus incomplete contracts occurs [58], thereby increasing the costs of contract performance.

**Fig 1:**
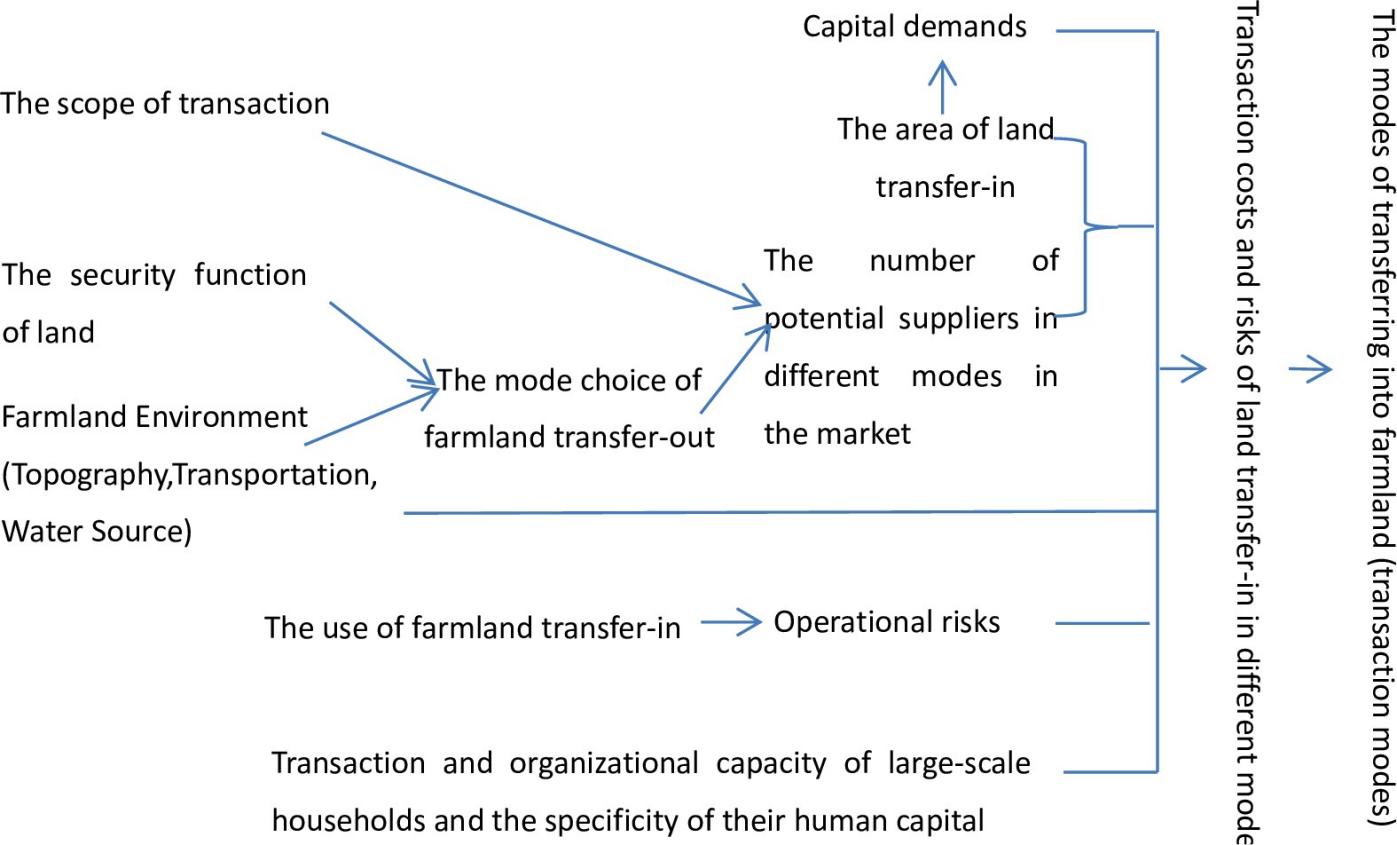
The mode choice of large-scale households’ farmland transfer-in. TCMR, lease and shareholding have different transaction costs under different conditions (the area and use of land transfer-in, the human capital of large-scale households, land environment, the security function of land, etc.), due to the differences in the scope of transaction, risk allocation, business decision-making rights allocation, the stability of contract, the allocation of changes in future land value, the impact on the security function of land, and the capital demand of the transferee. According to transaction cost economics, large-scale households will choose the mode with the lowest transaction cost to transfer into farmland. The figure is based on this logic.

In terms of the main dimensions of Williamson’s transaction dissimilarity, this paper analyzes transaction costs and the choice of three modes among TCMR, lease and shareholding from the perspective of large-scale households’ farmland transfer-in under different circumstances (Fig 1). These circumstances include circulation areas and uses, human capital characteristics of large-scale households, the environment of farmland and the security function of land.

### (1) Effects of areas and uses of circulation on the mode choice of farmland transfer-in

Due to the land system that rural land is collectively owned by farmers and only the collective members enable to enjoy contractual management rights of the collective land, the scope of TCMR is narrow and is limited to occur among the same collective members (See the Rural Land Contract Law of the People’s Republic of China). Meanwhile, TCMR will make the transferor lose the contractual rights and the security function of land, thus, only a few households are willing to choose TCMR to transfer out of land [59]. According to the statistics of the Ministry of Agriculture and Rural Affairs of the People’s Republic of China, the area of TCMR only accounts for 2.41% of the circulation area of the farmland contracted by rural households in China by 2020. Therefore, TCMR is difficult to meet the requirements of households’ largely transferring into farmland. However, lease and shareholding have been expanded beyond the collective, overcoming the narrow scope of TCMR. In terms of lease and shareholding, although the contractors transfer out of land management rights, they retain contractual rights. It makes a large number of contractors who are willing to transfer out of the contracted land but are unwilling to give up their contractual rights enter the market and the transferee can gather land in a large range. Consequently, ceteris paribus, with the increase of the areas of transfer-in, the probability of choosing lease and shareholding may increase relatively, whereas the probability of choosing TCMR may decrease relatively.

In addition, circulation areas may also affect the mode choice of farmland transfer-in through capital demand. The greater the areas of transfer-in (the scale of management), the greater the capital demand, and the more likely the transferee is to choose the contractual form which can reduce capital demand. Under the contracts of TCMR and the fixed rent contract, the transferee or lessee needs to pay the price of TCMR or rent, while it is not required to pay that price or rent under the shareholding contract. Therefore, ceteris paribus, in order to reduce the demand for funds, the probability of large-scale households’ choosing shareholding may increase faster than that of choosing lease with the increase of the areas of transfer-in.

The uses of farmland transfer-in include crop cultivation, garden and forestry management, breeding, etc. Different uses have different production cycles. The longer the cycle, the greater the uncertainty (For example, within the contract period, due to the laws or policies related to farmland circulation and the market price of farmland circulation may change, the transferor may request to terminate the contract under the pretext of the change of the agreed conditions of the contract.), and the higher the operational risks may be [60]. Therefore, the uses of farmland transfer-in may have an important influence in the mode choice of transfer-in owing to the differences in operational risks.

### (2) Effects of the characteristics of human capital of large-scale households on the mode choice

The characteristics of human capital of large-scale households include the ability to search for trading partners and information, organizational ability, and the specificity of human capital. Firstly, when large-scale households transfer into farmland, the costs and scope of searching for trading partners are affected by search capability. The stronger the search ability, the more likely they are to lease land or absorb others to share with land management rights beyond the collective, so the probability of choosing lease and shareholding may rise relatively. Secondly, from the perspective of management decision-making, the transferee or the lessee enjoys the exclusive management decision-making rights under the contracts of TCMR and lease. While, under the contract of shareholding, the costs of decision-making are more higher as shareholders collectively execute their decision-making rights [61, 62]. In order to reduce the costs of decision-making, shareholding requires large-scale households to have higher organization and control capability as the core subjects of the contract. Therefore, ceteris paribus, as large-scale households’ organization and control capability becomes stronger, the probability of their choosing shareholding when transferring into cultivated land may relatively increase. Thirdly, the stronger the specificity of large-scale households’ human capital dedicated to agriculture (such as cultivation experiences), the more likely they are willing to choose a stable and persistent contract to play the role of that human capital when transferring into farmland. The survey finds that so far, due to the imperfection, great uncertainty, low default cost and weak contract spirit of the market of farmland management right transfer, the lease and shareholding contracts have poor stability (Specifically, the period of lease is short (mostly less than 5 years), and the lessor’s request for raising rent or cancellation of the contract is common within the contract period. For shareholding contract, not only the costs of decision-making are high, but also the phenomenon that land shareholders require withdrawing shares occurs sometimes.) [63, 64]. Compared with lease and shareholding, the transfer of contractual management rights (TCMR) is the most stable and persistent [65]. Once TCMR occurs, the transferee stably obtains the contractual management rights of land. Therefore, ceteris paribus, the probability of choosing TCMR may relatively increase when large-scale households transfer into farmland with stronger specificity of human capital. This paper chooses gender, age, and educational attainment of household heads of large-scale households and the number of labor forces to reflect their characteristics of human capital.

### (3) Effects of the environment of farmland on the mode choice of land transfer-in

The environment of cultivated land includes topography, traffic, irrigation water and so forth, which may have multiple effects on the mode choice of farmland transfer-in. First, ceteris paribus, the contractors’ willingness to transfer out of the contractual management rights may be relatively lower in areas with better environmental conditions, such as plains, traffic-developed areas and water-sourced areas. That is, for the transferee, the number of potential counterparties choosing TCMR is relatively smaller, and the costs of searching and waiting for transaction are relatively higher. Thus, subject to this, the probability of large-scale households’ choosing TCMR to transfer into farmland may be reduced relatively. However, there may also be an opposite forces, that is, for land with better environmental conditions, the transferee may relatively prefer to establish a stable and persistent contractual relationship. While as mentioned above, compared with lease and shareholding, TCMR is the most stable and lasting. Therefore, the probability of choosing TCMR may relatively increase. Subsequently, environmental conditions may affect the future value of cultivated land. Nevertheless, due to the complexity and uncertainty of environmental factors that affect the future value of land and the bounded rationality of human beings, it is fairly difficult to predict the future value of land, and the costs of finding transaction prices and bargaining may be quite high in TCMR and lease. In the shareholding, equity returns automatically reflect the change of land future value, thus saving the costs of predicting the change of land future value. As a result, ceteris paribus, the more complex and uncertain the environmental factors that affect the future value of land are, the more likely large-scale households are to choose shareholding when they transfer into farmland. Therefore, the influence of environment of cultivated land on the mode choice of transfer-in is uncertain and needs to be tested.

### (4) Effects of the security function of land on the mode choice of farmland transfer-in

As mentioned above, large-scale households’ mode choices of transferring into cultivated land are subject to the number of potential counterparties with different modes in the market, namely households’ mode choices of transferring out of cultivated land. The land security function (Although there are different opinions on the security function of rural land, the land does bear different levels of social security functions for most farmers since the social security system is not perfect and the security level is low in Rural China. Many elderly farmers rely on land management to make up for the lack of pension. Land management is also the employment guarantee for a large number of farmers who have transferred employment after the failure of transfer employment [68–70].) is an important factor that influences households’ decision-making. Meanwhile, under different circulation modes, land has different security function to the contractors. For instance, if contractors choose TCMR, they will completely lose the land security function. Although lease or shareholding will weaken the security function of land to contractors, it will not entirely make them lose the security function of land (First, as the lease term expires or enterprises where contractors take shares are dissolved, the land management rights will be returned to the contractors. At this time, if necessary, the contractors enable to choose farming by themselves to achieve the survival and employment security function of land. Second, the rent or dividend income also provides a certain survival guarantee for contractors.). Consequently, the land security function may have an important impact on households’ mode choices of transferring out of cultivated land and then on that of transferring into cultivated land. In addition, in terms of regions, the land security function mainly depends on the development level of non-agricultural industries and the rural social security and so forth. Ceteris paribus, the more developed the non-agricultural industries and the rural social security are, the weaker the security function of land is, and thus the more likely farmers are to choose TCMR when they transfer out of cultivated land. As a result, for the transferee, the probability of choosing TCMR may increase accordingly.

## 4. Data sources, variable setting and statistical description of samples

The data used in this paper are from the public sample of the Third National Agricultural Census of the People’s Republic of China (The data used in this paper are from the public sample database of the third national agricultural census data. This paper is supported by data from the National Bureau of Statistics and the National Bureau of Statistics—Tsinghua University Data Development Center. Besides, the views and results of this paper only represent the views of the author, not the views or opinions of the data development center or the National Bureau of Statistics.). As shown in Table 1, the total sample is 14126, covering large-scale households from 41 prefectures in 22 provinces.

**Table 1:** Variable description and statistical characteristics.

Variable	Value	Freq.	Percent	Mean	Std. Dev.	Min	Max
**Mode**	TCMR = 0	244	1.73				
	Lease = 1	13670	96.77				
	Shareholding = 2	212	1.5				
**Areas**	Hectare			7.2521	15.4928	0.0067	866.6667
**Uses**	Crop = 1	12311	87.15				
	Garden & forestry = 2	792	5.61				
	Breeding = 3	951	6.73				
	Others = 4	71	0.5				
**Gender**	Female = 0	1057	7.48				
	Male = 1	13069	92.52				
**Age**	years			47.1659	11.2909	1	94
**Education**	Unedu = 1	202	1.43				
	Primary = 2	3676	26.02				
	Junior = 3	8550	60.53				
	Senior & above = 4	1698	12.02				
**Labor forces**				2.7114	1.0328	0	12
**Topography**	average			1.6534	0.5308	1	3
**Traffic**	average			0.3995	0.325	0	2
**Water**	average			0.9403	0.0876	0.3538	1
**RPI**	%			43.5718	7.711	15.0008	87.4503
**RFI**	%			11.4974	4.8637	0.4	24

The statistical characteristics of the data are the results of the authors’ collection and calculation according to the public sample of the Third National Agricultural Census of the People’s Republic of China.

In this paper, the explained variable is the mode of large-scale households’ farmland transfer-in: TCMR = 0, lease = 1, shareholding = 2. In the sample, 96.77% of large-scale households transfer into land by lease, with 1.73% by TCMR and 1.50% by shareholding.

The explanatory variables include circulation areas, uses, and human capital of large-scale households.

### (1) Circulation areas

The average households’ areas of transfer-in in the sample are about 7.25 hectares, with the minimum of 0.0067 hectares and the maximum of 866.67 hectares.

### (2) Circulation uses

The most majority of large-scale households transfer into cultivated land for crop cultivation, accounting for about 87.15 percent of the total sample, only 6.73 percent for breeding and 5.61 percent garden & forestry managements. In addition, few used for other business.

### (3) The characteristics of human capital of large-scale households

This paper chooses gender, age, educational attainment of the household heads and the number of labor forces to reflect the characteristics of human capital of large-scale households. Among the samples, 92.52% household heads are male and 7.48% are female. The average age of the household heads is about 47 years old. The smallest is 1 year old, and the largest is 94 years old. Moreover, the education attainment of household heads is mainly junior high school, accounting for 60.53%, followed by primary school, and high school & above education accounting for 26.02% and 12.02% respectively. Additionally, few have not attended school and the average number of labor forces per household is about 2.7, with the minimum of 0 and the maximum of 12.

Control variables include village topography (plains = 1; hilly areas = 2; mountainous areas = 3), township traffic conditions (whether there are highway exits and entrances, train stations, docks: none = 0; one of the three = 1; two of the three = 2; all of the three = 3), the main irrigation water source of the village (no = 0; yes = 1), the ratio of pension insurance coverage (the ratio of pension insurance coverage to village household registration population, namely RPI), and the proportion of output value of the primary industry of the province (city) taking up in GDP (RFI, hereinafter referred to as the proportion of the primary industry). Among them, the first three variables reflect the environmental conditions of cultivated land, and the last two variables reflect the security function of local land.

Owing to the database used in this paper have been desensitized, it is impossible to identify or infer which administrative village or township corresponds to the large-scale households in the sample, but it can identify whether they belong to the same prefecture-level city. Therefore, this paper uses the prefecture average level of topography, traffic, water and RPI among those control variables.

## 5. Empirical analysis

### 5.1. Model setting

The explained variable contains three unordered categories (TCMR = 0; lease = 1; shareholding = 2), so this paper adopts *mlogit* regression. According to the above theoretical analysis, the *mlogit* model is established as follows:

$$ML=\beta_{0}+\sum_{i=1}^{n}\beta_{i}x_{i}+\sum_{i=1}^{n}\delta_{i}z_{i}+\mu$$ (1)

In function (1), *ML* indicates the *logit* predicted value or Log Odds of households choosing different modes of farmland transfer-in. *x_i_* represents each explanatory variable; *z_i_* represents each control variable; *μ* is a random error item; *β*_0_, *β_i_, δ_i_* are parameters to be estimated.

Since the circulation areas have abnormal observations, the logarithm of circulation areas is taken in the model.

### 5.2. Results and discussions

Using STATA for statistical analysis, *mlogit* regression analysis is carried out on the data with the minimum value as reference and the results are shown in Table 2. Moreover, In order to deal with the heteroscedasticity in the linear probability model, Table 2 reported robust standard error and statistics, and Prob > chi2 = 0.0000 indicates that overall explanatory variables and control variables have significant impacts on explained variables.

**Table 2:** Model regression results.

Mode	Model 1	Model 2	Model 3	Model 4
0 = TCMR	(base outcome)			
**1 = lease**				
**ln (areas)**	0.2735*** [0.0496]	0.2750*** [0.0503]	0.2791*** [0.0501]	0.2794*** [0.0502]
**Uses**				
**2 = Garden & forestry**	0.0498 [0.2992]	0.0448 [0.3040]	0.0704 [0.3043]	0.0707 [0.3045]
**3 = Breeding**	-0.1271 [0.2270]	-0.1890 [0.2317]	-0.1673 [0.2313]	-0.1655 [0.2314]
**4 = Others**	-0.5114 [0.6348]	-0.6067 [0.6304]		
**Male**	0.3253 [0.2125]	0.2926 [0.2157]	0.3100 [0.2164]	0.3412 [0.2130]
**Edu**				0.1839* [0.1083]
**2 = Primary**	0.6877* [0.3562]	0.5962* [0.3411]	0.5619* [0.3431]	
**3 = Junior**	0.7287* [0.3993]	0.6482* [0.3861]	0.5998* [0.3874]	
**4 = Senior & above**	0.7406** [0.3619]	0.6896** [0.3485]	0.6940** [0.3504]	
**Age**	-0.0100* [0.0058]	-0.0123** [0.0055]	-0.0137*** [0.0053]	-0.0125** [0.0056]
**Labor**	-0.1303** [0.0582]	-0.1408** [0.0569]	-0.1468*** [0.0566]	-0.1482*** [0.0567]
**(age-mean)#(labor-mean)**		0.0072* [0.0039]	0.0082** [0.0038]	0.0089** [0.0040]
**Topography**	-0.4129*** [0.1252]	-0.3844*** [0.1214]	-0.3731*** [0.1223]	-0.3718*** [0.1220]
**Traffic**	0.3635 [0.2459]	0.2910 [0.2451]	0.2604 [0.2456]	0.2557 [0.2447]
**Water**	-3.5972*** [0.9420]	-3.4412*** [0.9535]	-3.4743*** [0.9682]	-3.4805*** [0.9665]
**RPI**	-0.0254*** [0.0088]	-0.0201** [0.0102]	-0.0224** [0.0102]	-0.0227** [0.0102]
**RFI**	0.0756*** [0.0188]	0.0753*** [0.0211]	0.0762*** [0.0212]	0.0770*** [0.0211]
**_cons**	7.9926*** [1.1708]	7.4562*** [1.1904]	7.6823*** [1.2015]	8.0927*** [1.2075]
**2 = Shareholding**				
**ln(areas)**	0.5256*** [0.1477]	0.5763*** [0.1550]	0.5804*** [0.1549]	0.5632*** [0.1533]
**Uses**				
**2 = Garden & forestry**	1.7166** [0.7062]	1.9695*** [0.7376]	1.9947*** [0.7378]	1.9047*** [0.7234]
**3 = Breeding**	0.3162 [0.8269]	0.4745 [0.8513]	0.4958 [0.8511]	0.4659 [0.8427]
**4 = Others**	-2.9468*** [0.7437]	-2.6276*** [0.7381]		
**Male**	1.1193 [0.9097]	1.0567 [0.9287]	1.0738 [0.9288]	1.1385 [0.9298]
**Edu**				1.1507** [0.5188]
**2 = Primary**	2.7996*** [0.8614]	2.2042*** [0.8242]	2.2096*** [0.8249]	
**3 = Junior**	2.8103*** [1.2333]	2.2648*** [0.8330]	2.2183*** [0.8155]	
**4 = Senior& above**	3.7307*** [0.7654]	3.1551*** [0.8169]	3.1225*** [0.9323]	
**Age**	-0.0414** [0.0194]	-0.0335** [0.0169]	-0.0350** [0.0168]	-0.0266** [0.0129]
**Labor**	-0.2450 [0.2066]	-0.3054 [0.2088]	-0.3113 [0.2087]	-0.3422 [0.2134]
**(age-mean)#(labor-mean)**		0.0045** [0.0022]	0.0046** [0.0022]	0.0044** [0.0020]
**Topography**	-0.0964 [0.4989]	-0.1358 [0.4884]	-0.1248 [0.4886]	-0.1231 [0.4897]
**Traffic**	1.0665** [0.5285]	1.3131** [0.5368]	1.2828** [0.5370]	1.2655** [0.5309]
**Water**	-2.1282 [2.1879]	-1.2142 [2.8163]	-1.2472 [2.8213]	-1.4400 [2.7643]
**RPI**	-0.0158 [0.0248]	-0.0288 [0.0297]	-0.0311 [0.0297]	-0.0322 [0.0291]
**RFI**	-0.0106 [0.0542]	-0.0151 [0.0146]	-0.0150 [0.0146]	-0.0157 [0.0153]
**_cons**	-14.9939*** [2.8576]	-10.4918*** [3.6347]	-10.2699*** [3.6388]	-10.2714*** [3.6582]
**Number of obs**	14126	14126	14055	14055
**Wald chi2(0)**	3459.81	3374.19	3113.27	3152.25
**Prob > chi2**	0.0000	0.0000	0.0000	0.0000
**Pseudo R2**	0.1657	0.1752	0.1762	0.1737
**Log pseudolikelihood**	-1302.0784	-1288.7724	-1274.9939	-1278.3331

Note: In Table 2, Coef. and Robust Std. Err. are reported.

*, * *, * * * represent 10%, 5% and 1% significance levels respectively.

The original data for regression is sourced from the public sample of the Third National Agricultural Census of the People’s Republic of China.

This paper also examines the interaction effect between explanatory variables. In order to avoid multicollinearity, a middle strategy is used for interaction items (except for virtual variables and less classified categorical variables). The results show that there is a significant interaction effect only between the age of household heads and the number of labor forces. Therefore, the interaction term of age and labor, namely (*age-mean)#(labor-mean)*, is introduced into Model 2. The impacts of these variables are discussed as follows.

#### 5.2.1. Effects of areas and uses of circulation on the mode choice of farmland transfer-in

On one hand, the effects of circulation areas are discussed as follows. Ceteris paribus, with the increase of areas of circulation, the probability for large-scale households to choose lease and shareholding increases significantly and the probability of the latter increases faster, which is consistent with the previous theoretical analysis. When large-scale households’ circulation areas double, the Log Odds of choosing lease and shareholding increased by 0.2735 and 0.5256 at a significance level of 1% respectively (Model 1).

One the other hand, the following is the effect of circulation uses on the mode choice. In contrast to large-scale households who transfer into cultivated land to operate crop cultivation, for those whose purposes are garden and forestry, the Log Odds of choosing lease of is relatively higher, but it is insignificant. Log Odds of choosing shareholding increases by 1.7166 at the 5% significance level (Model 1), that is, large-scale households prefer to choose shareholding. This may be mainly because that garden & forestry have longer business cycle, greater uncertainty and higher operational risks compared with crop cultivation, and large-scale households tend to choose shareholding contracts to decentralize risk when they transfer into cultivated land. In addition, compared with those whose purpose is crop cultivation, Log Odds of choosing lease decreases relatively when large-scale households’ purpose is breeding, and Log Odds of choosing shareholding increases relatively, but they are insignificant.

#### 5.2.2. Impacts of gender and educational attainment of household heads on the mode choice

First, in contrast to large-scale households with female household heads, for those with male household heads, the probability of choosing lease and shareholding is relatively higher in transferring into cultivated land, but both are insignificant.

Second, with the improvement of household heads’ educational attainment, the probability of choosing lease and shareholding increases, but the latter increases faster. Compared with those uneducated, Log Odds of large-scale households whose household heads have primary school, junior high school, senior high school & above diplomas choosing to lease increases by 0.6877 at the 10% significance level, 0.7287 at the 10% significance level and 0.7406 at the 5% significance level, respectively. Log Odds of large-scale households’ choosing to share increases by 2.7996, 2.8103 and 3.7307 (Model 1) at 1% significance level, respectively. It is probably because that cross-collective lease and shareholding require higher ability to search for trading partners and information, communicate, and negotiate, as well as supervise and execute contracts in contrast to the transfer of contractual management rights (TCMR) among members within the collective. Additionally, compared with TCMR and lease contracts, shareholding contracts have higher requirements on organization and control capability of the core subjects, whereas education significantly improves the ability of the educated in these aspects. Therefore, when households transfer into cultivated land, households’ educational attainment has a significant positive impact on the selection of lease and shareholding, but the impact on shareholding is more greater.

#### 5.2.3. Effects of age and number of labor forces on the mode choice and their interaction effects

Ceteris paribus, with the increase of the age of household heads, the probability of large-scale households’ choosing lease and shareholding when transferring into cultivated land decreases significantly, but the latter decreases faster. In other words, the probability of choosing TCMR increases relatively. The followings are two possible reasons for that. One reason is that older farmers have less opportunities for non-agricultural employment, but own stronger specificity of their human capital and more farming experience dedicated to agriculture than younger farmers. Consequently, they pay more attention to the stability of contracts when they transfer into cultivated land. As mentioned above, at present, the contracts of TCMR are more stable and durable than lease and shareholding contracts in China. Besides, for shareholding contracts, farming experience is not included in the surplus distribution, which further restricts older farmers with more farming experience to share with land management rights. Therefore, when large-scale households transfer into cultivated land, the negative impact of household heads’ age on the selection of shareholding is greater than that on lease.

According to the interaction term between the age of household heads and the number of family labor forces in Model 2, for large-scale households with different numbers of labor forces, the impact of household heads’ age on the mode choice of transferring into cultivated land has significant differences. In terms of the average number of family labor forces (about 3), for one year increase in household heads’ age, Log Odds of choosing lease and shareholding decrease by 0.0123 and by 0.0335 at the 5% significance level respectively. The coefficient of interaction term is positive, indicating that the increase in the number of family labor forces weakens the negative impact of household heads’ age on the selection of lease and shareholding. In other words, the household heads’ age has a negative impact on choosing lease and shareholding, but this negative impact is less for families with more labors than those with less labors.

In the light of the choice of TCMR and lease, the number of family labor forces has a significant negative impact on the selection of lease. From the interaction term in Model 2, the impact of the number of family labor forces is significantly different for large-scale households with different household heads’ age. At the average age of household heads about 47 years old, for each additional family labor forces, Log Odds of the selection of lease decreases by 0.1408 at the 5% significance level. In addition, the coefficient of interaction term is positive, indicating that the increase of household heads’ age weakens the negative impact of the number of family labor forces on the selection of lease. In other words, the number of family laborers has a negative impact on choosing lease, but the negative impact on households with older household heads is less than those with younger household heads. From the perspective of the selection of TCMR and shareholding, the probability of choosing shareholding also shows a downward trend with the increase of the number of labor forces, but it is insignificant.

#### 5.2.4. Effects of control variables on the mode choice of land transfer-in

*5*.*2*.*4*.*1*. *Effects of topography*. Log Odds of choosing lease decreases by an average of 0.4129 at the 1% significance level in accordance with the order of plains, hilly areas, and mountainous areas (model 1). On the contrary, the probability of choosing TCMR increases in turn in the same order. This may be because the worse the topography conditions, the higher the households’ willingness to transfer out of contractual management rights, which brings about more suppliers of TCMR. That is, for the transferee, through TCMR, the costs of searching and waiting for in the transaction decrease relatively. Accordingly, when large-scale households transfer into cultivated land, the probability that they choose TCMR increases relatively. Additionally, there is also a decreasing trend to choose shareholding in accordance with the order of plains, hilly areas, and mountainous areas, but it is insignificant.

*5*.*2*.*4*.*2*. *Impacts of traffic conditions*. In terms of the choice of TCMR, lease and shareholding, with the improvement of traffic conditions, the probability of choosing lease increases relatively, but it is insignificant and the probability of choosing shareholding increases significantly at the 5% significance level. Log Odds of choosing shareholding increases by 1.0665 on average as traffic conditions improve by per one level. It is probably due to the fact that the better the traffic conditions, the greater the space for the rise of land value. But it is difficult to predict the future value of land, and the costs of finding transaction price and bargaining in TCMR and lease may be high. However, from the perspective of shareholding, the return on equity of shareholders automatically reflects future changes in land value, saving the costs of predicting that changes.

*5*.*2*.*4*.*3*. *Impacts of water sources*. Between the choice of TCMR and lease, compared with regions without water sources, Log Odds of lease in regions with water sources decreases by 3.5972 at the 1% level (Model 1). This may be because ceteris paribus, the costs of irrigation in regions with water sources are lower, which makes the transferee prefers to choose a more stable way to transfer into farmland with water sources. As mentioned above, in contrast to lease contracts, TCMR is more stable and persistent. Furthermore, Log Odds of choosing shareholding in regions with water sources also shows a downward trend compared with regions without water sources, but it is insignificant.

*5*.*2*.*4*.*4*. *Impacts of the ratio of pension insurance coverage*. From the perspective of the choice of TCMR and lease, for every 1% increase in the ratio of pension insurance coverage, Log Odds of choosing lease decreases by 0.0254 when large-scale households transfer into cultivated land at the 1% significance level (Model 1). That is, with the increase in the ratio of pension insurance coverage, the probability of choosing TCMR increases relatively. It is probably because that ceteris paribus, the higher the ratio of pension insurance coverage, the smaller the security function of land, and thus the higher the probability for households to transfer out of the contracted land, the more the transferor. That is, for the transferee, the more potential counterparties to choose TCMR, the lower the costs of searching and waiting for the transaction. As a result, the probability of choosing TCMR is relatively higher when transferring into cultivated land. Between the choice of TCMR and shareholding, with the increase in the coverage rate of pension insurance, Log Odds of shareholding also decreases, but it is insignificant.

*5*.*2*.*4*.*5*. *Impacts of the proportion of primary industry*. Between the choice of TCMR and lease, when the proportion of primary industry increases by per 1%, Log Odds of choosing lease increases 0.0756 at the 1% significance level (Model 1). That is, with the increase in the proportion of primary industry, the probability of choosing TCMR is reduced. This may be because the higher the proportion of primary industry, the more backward the secondary and tertiary industries, which brings about more stronger land security function. According to this, when the contractor transfers out of the cultivated land, the probability of choosing TCMR and lease relatively decreases and increases respectively. Namely, for the transferee, the potential counterparties of TCMR relatively reduce, and the cost of searching and waiting of TCMR relatively increases. When the transferee chooses lease, the potential counterparties increase and the cost of searching and waiting decreases relatively. Therefore, the probability of choosing lease is relatively higher when transferring into cultivated land. However, the influence of the proportion of the primary industry on the selection of TCMR and shareholding is insignificant.

### 5.3. Robustness test

Considering that models may have endogenous problems and the results are not robust, in this paper, we carry out the robustness tests in model 3 and model 4. On the one hand, according to Lu & Wang’s [66] practice of removing special samples, the special samples of transferring into cultivated land for other purposes including multiple uses are excluded in Model 3. The results show that in contrast to transferring into cultivated land for crop cultivation, the probability of those whose purpose is garden & forestry management choosing lease is relatively higher, but it is insignificant, and the possibility of choosing shareholding increases significantly. These correspond to the results of Model 1 and Model 2. Meanwhile the symbols of other variables are also consistent with those of Model 1 and Model 2, and the changes of coefficient and significance are little.

On the other hand, in Model 4, the educational attainment of the heads of large-scale households is treated as a continuous variable. It is shown that among the choice of three circulation modes, the probability of choosing lease and shareholding increases significantly with the improvement of educational attainment, while the latter increases faster, which is consistent with the results of Model 1 and Model 2. In addition, the symbols of other variables also correspond to those of Model 1 and Model 2, and the coefficient and significance change little. Consequently, in this study, the estimation results are robust.

## 6. Conclusions

Based on the contract theory and transaction cost economics, this paper adopts the public samples of the Third National Agricultural Census of People’s Republic of China, and takes the case that large-scale households transfer into farmland to analyze the choice of three basic circulation modes which include the transfer of contractual management rights (TCMR), lease and shareholding. According to the contract theory and transaction cost economics, saving the transaction cost is the basic principle of contract choice. TCMR, leases and shareholding have different characteristics in terms of the scope of transaction, land security function, market supply, capital demands, transaction costs and risks; and thus have different transaction costs and risks under different conditions such as the area of transfer-in, the use, the human capital of the parties, the number of laborers, and farmland environment (see Part 3 for details). Therefore, under different conditions, the transfer subjects have different tendencies on the mode choice of land transfer namely contract form. The followings are the findings of our study.

On the one hand, when large-scale households transfer into cultivated land, the areas and uses of circulation, the number of family labor forces and characteristics of household heads have important impacts on the selection of the mode of circulation. First, with the increase of circulation areas and improvement of educational attainment of household heads, the probability of choosing lease and shareholding increases relatively, but the latter increases faster. Subsequently, in contrast to large-scale households whose transfer purpose is crop cultivation, those whose transfer purpose is garden and forestry management are more likely to choose shareholding. Furthermore, there is an interaction effect between the age of household heads and the number of family labor forces on the mode choice of circulation. Specifically, the probability of choosing lease and shareholding shows a downward trend as household heads’ age increases, but the latter decreases faster, and this trend weakens with the increase of the number of family labor forces. With the increase of the number of family labor forces, the probability of choosing lease also presents a downward trend which weakens gradually with household heads’ age increasing.

On the other hand, the mode choice of large-scale households’ transferring into cultivated land is also significantly affected by local topography, irrigation, traffic conditions, industrial structure and social security development. First, from the perspective of topography, according to the order of plains, hilly areas and mountainous areas, the probability of choosing lease decreases significantly, and the probability of choosing TCMR increases in turn. Subsequently, in the light of irrigation conditions, large-scale households in areas with water sources prefer to choose TCMR, and the probability of choosing lease significantly reduces. Moreover, the probability of choosing shareholding shows a marked increase in areas with better traffic conditions. Additionally, as the proportion of primary industry rises, the probability of choosing lease increases significantly, and the probability of choosing TCMR is reduced relatively. Ultimately, as the ratio of pension insurance coverage increases, the probability of choosing lease decreases significantly, and the probability of choosing TCMR increases relatively.

## 7. Policy implications

Based on the above research findings, five policy implications are put forward as follows. First, saving transaction costs is the basic principle of contract selection. Different circulation modes have different transaction costs under the conditions of different areas and uses of circulation, human capital and number of labor force. Generally speaking, both the transferor and transferee pretty understand that conditions. Therefore, the mode choice of farmland transfer must adhere to circulation parties’ independent choice and negotiation to stimulate the initiative and creativity of them. In addition, mandatory orders and one-size-fits-all policy should be inhibited. Second, for the selection of circulation modes, the government and rural grassroots organizations should play a main role in educating, training, demonstrating and guiding farmers by providing them with necessary knowledge to choose circulation modes, such as the characteristics and adaptability of different circulation modes. Moreover, it is important for government and rural grassroots organizations to help farmers to obtain information, provide negotiation platform, analyze the characteristics of supply and demand of different circulation modes, and discuss the possible circulation modes from the ability to save transaction costs and then put forward suggestions. Meanwhile, the government should support the development of rural land rights value assessment institutions through fiscal, taxation and credit policies to reduce the costs of discovering transaction prices and bargaining in land rights transactions. Third, improve the quality of rural education and develop vocational education and skills training. Thus, that will enhance farmers’ search capability for transaction partners and information, communication and negotiation, supervision and implementation of contracts to promote the circulation of land management rights. Fourth, support the development of rural transport and play a positive role of it in land lease and shareholding. Specifically, strengthen village road construction; improve the transportation network; increase the density of highway exits and entrances, railway stations and docks. Furthermore, it is extraordinarily possible to try to achieve a scene where there are entrances and exits for villages and towns along the expressway, docks for villages and towns along the waterway, and railway stations for villages and towns with a certain population along the railway. Fifth, it is necessary to reduce farmers’ dependence on land. Specifically, improve the level of rural social security; increase the employment of farmers and then promote the transfer of rural labor force by developing the processing and circulation of agricultural products, rural leisure tourism, rural service industry and so on. Additionally, water conservancy construction of cultivated land should be strengthened to solve the problem of irrigation water source in water-free areas. Accordingly, these measures will play a positive role in TCMR.

## 8. Contribution, limitations and expectation

Regarding the study of rural land rights transfer or configuration modes, the contribution of this paper is mainly reflected in two aspects. First of all, it expands the research field of rural land transfer, turning the focus to the choice of specific transfer modes, like TCMR, lease and shareholding. Taking large-scale households’ land transfer-in as an example, this paper investigates the effect of the area and use of transfer-in, labor resources and human capital of households, farmland environment and land security function on the mode choice of land transfer-in. Secondly, the different modes of land rights transfer are regarded as different forms of contracts, and the analysis paradigm of transaction cost economics is applied to the analysis of the mode choice of farmland transfer-in for large-scale households in this study. This study partly reveals the rule that the parties of rural land rights transfer choose the modes of transfer, and provides a reference for the parties with different characteristics under different conditions to choose the most efficient mode of transfer, and also for the government and rural grassroots organizations to play a role.

However, this study has some limitations due to the restriction of data resource. First, some factors that may have an impact on the mode choice of farmland transfer-in are not included in the analysis, like the local agricultural insurance and the financing environment. Second, the classification about the uses of farmland transfer-in is relatively rough. Third, the indicators reflecting the security function of farmland and land environment are not accurate enough since this paper uses regional averages or provincial data.

Therefore, this study can be further broadened from the following aspects. Firstly, a comprehensive analysis of the factors that affect the mode selection of farmland transfer-in could be considered in the future research. Secondly, environmental factors could be more accurate. In addition, future studies can focus on the mode choice of rural land rights transfer from the perspective of agricultural enterprises, farmers’ cooperatives, and the party who transfers out of land.
